# Epstein Barr Virus-positive large T-cell lymphoma presenting as acute appendicitis 17 years after cadaveric renal transplant: a case report

**DOI:** 10.1186/1752-1947-5-5

**Published:** 2011-01-12

**Authors:** Shiva K Ratuapli, Shishir Murarka, Karen A Miller, James C Ferraro, Haider Zafar

**Affiliations:** 1 Department of Medicine, Banner Estrella Medical Center, 9201 W. Thomas Road, Phoenix, AZ 85037, USA; 2 Department of Pathology, Banner Estrella Medical Center, 9201 W. Thomas Road, Phoenix, AZ 85037, USA

## Abstract

**Introduction:**

The majority of post-transplant lymphoproliferative disorders in renal transplant patients are of the B-cell phenotype, while the T-cell phenotype is rare. We report a case of Epstein Barr Virus-positive, T-cell lymphoma in a renal transplant patient, presenting unusually as acute appendicitis.

**Case presentation:**

A 45-year-old Hispanic male renal transplant patient presented with right-side abdominal pain 17 years after transplant. The laboratory studies were unremarkable. Laparoscopic exploration showed an inflamed appendix so a laparoscopic appendectomy was performed. Pathology of the appendix showed large cells positive for CD3, CD56 and Epstein Barr Virus-encoded RNA staining, and negative for CD20 and CD30. The tissue tested positive for T-cell receptor gene rearrangement by polymerase chain reaction analysis. Treatment management involved reduction of immunosuppression and initiation of chemotherapy with cisplatin, etoposide, gemcitabine, and solumedrol followed by cyclophosphamide, hydroxydaunorubicin, vincristine and prednisone). He recovered and the allo-grafted kidney is fully functional.

**Conclusion:**

We report a rare case of post-renal transplant large T-cell lymphoma, with an unusual presentation of acute appendicitis and Epstein Barr Virus-positivity, which responded well to chemotherapy.

## Introduction

Solid organ transplantation has been increasingly performed in recent years with the use of highly potent immunosuppressive agents to avoid rejection by the host. Post-transplant lymphoproliferative disorders (PTLD) are well known malignancies found in transplant patients, with an incidence reportedly 28 to 49 times greater than in the general population [1]. PTLD in renal transplant patients is reported to be higher in the paediatric population (10.1%) than the adult population (1.2%) [2]. PTLD in renal transplant patients was first described as a complication with azathioprine-based therapy [3], but was later described after therapy with multiple more novel immunosuppressive agents.

While the majority of PTLD in renal transplant patients are of the B-cell phenotype, a few exhibit the T-cell phenotype [4]. We report a case of Epstein Barr Virus (EBV)-positive T-cell lymphoma in a patient, who underwent cadaveric renal transplant 17 years ago and was on chronic multi-drug immunosuppression. Our patient presented unusually with abdominal pain and acute appendicitis.

## Case presentation

A 45-year-old Hispanic male who underwent cadaveric renal transplant in the right lower quadrant 17 years earlier, presented to the hospital with a three-month history of generalized abdominal pain with localization to the right side for two weeks. He was on chronic immunosuppression with tacrolimus, azathioprine, sirolimus and prednisone. The pain was more pronounced in the right upper quadrant, and the ultrasound imaging of the abdomen was suggestive of cholecystitis. Laboratory studies did not reveal any abnormalities. He could not confirm if he had had any problems or surgeries on his gall bladder. Hence, he underwent laparoscopic exploration of the gall bladder fossa. During surgery, adhesions of the omentum were found in the gall bladder fossa in the absence of the gall bladder, and an inflamed appendix was found elevated due to the transplanted kidney in the right lower quadrant. Laparoscopic appendectomy was performed and the tissue underwent pathological examination. He was discharged after an uneventful post-operative course.

**Figure 1:**
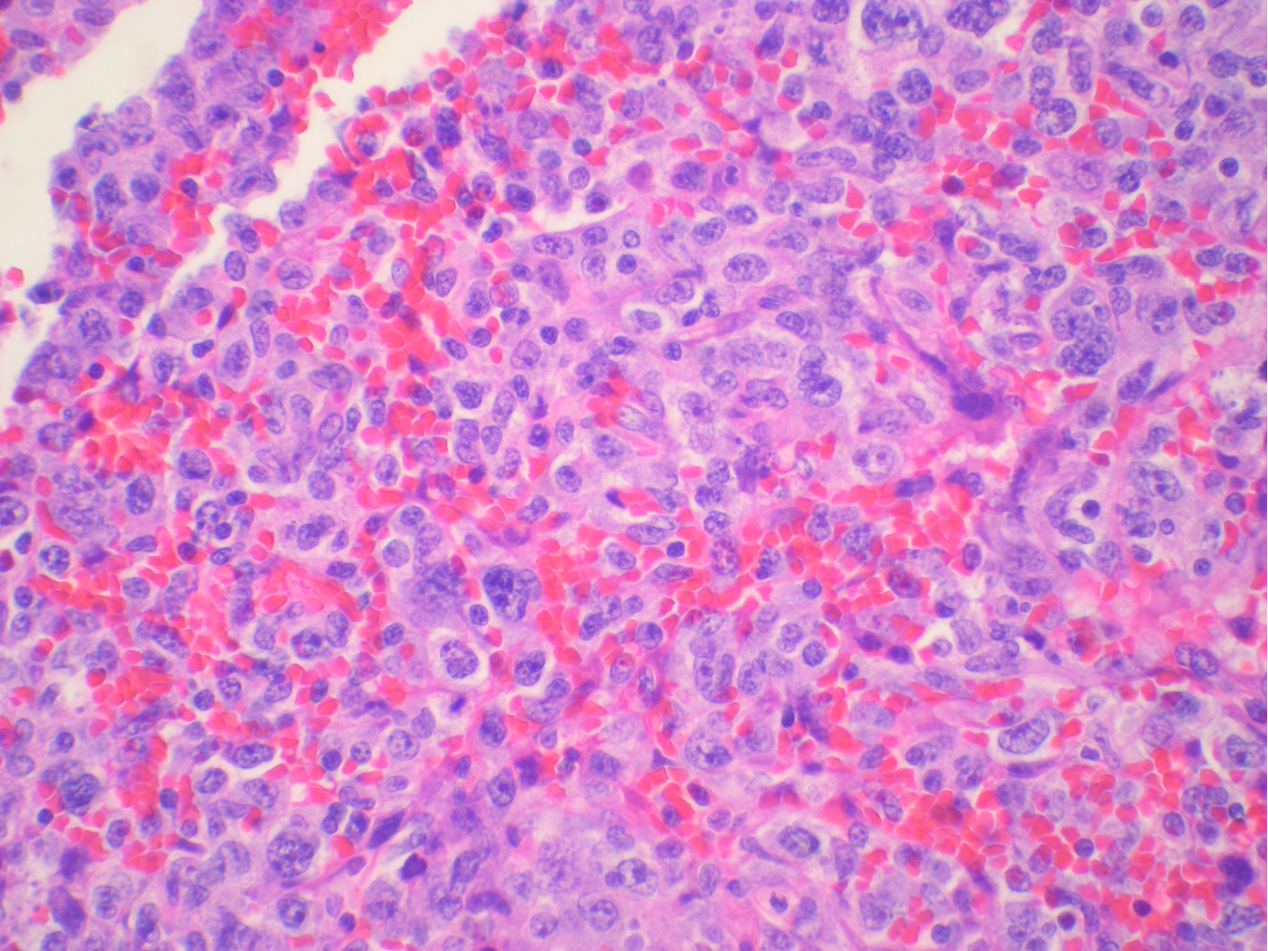
**Appendix biopsy showing large, pleomorphic lymphocytes with irregular nuclear contours and large nucleoli. (400 X)**.

**Figure 2:**
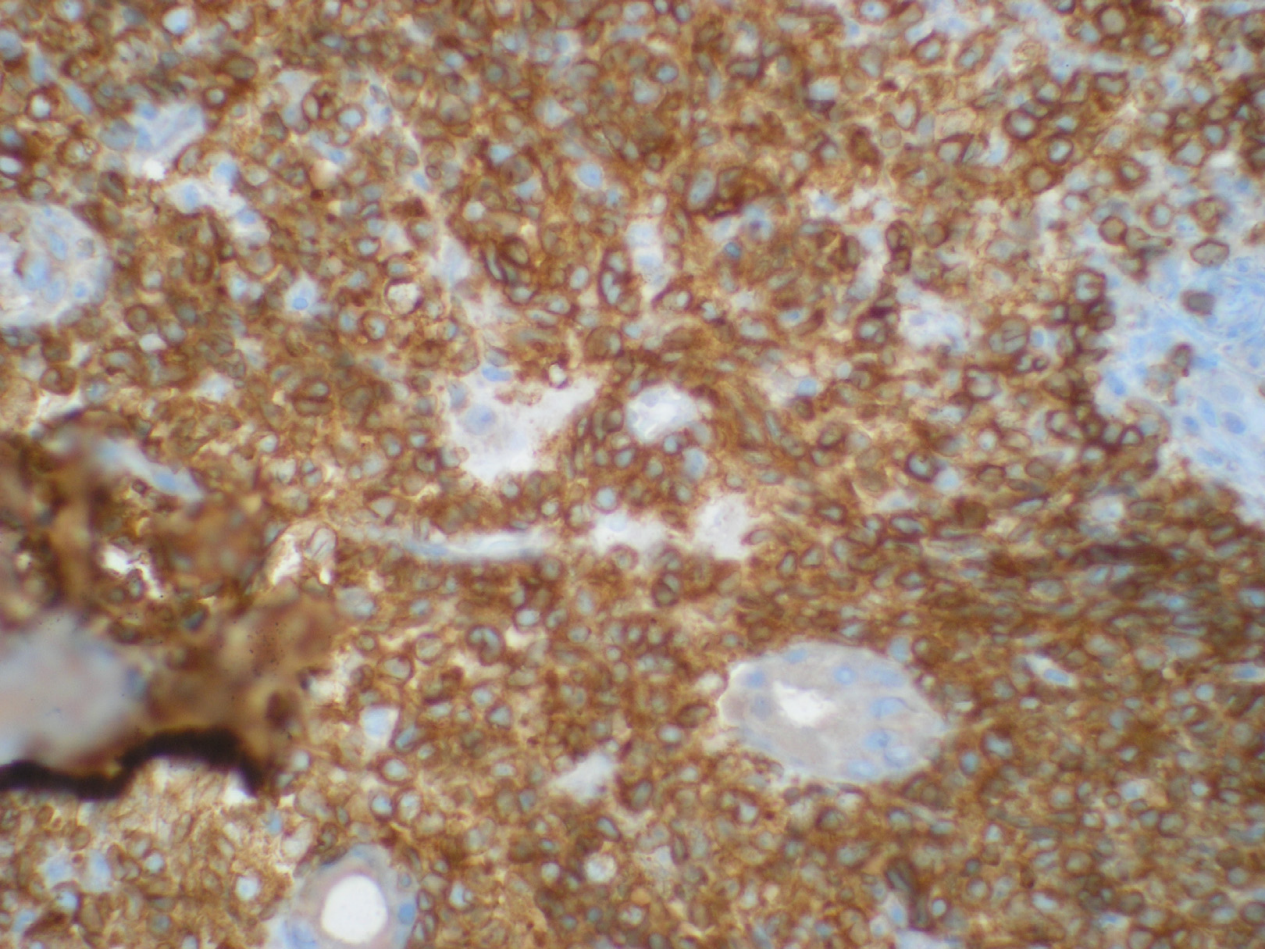
**Positive staining of lymphoid infiltrate for CD3 (400 X)**.

**Figure 3:**
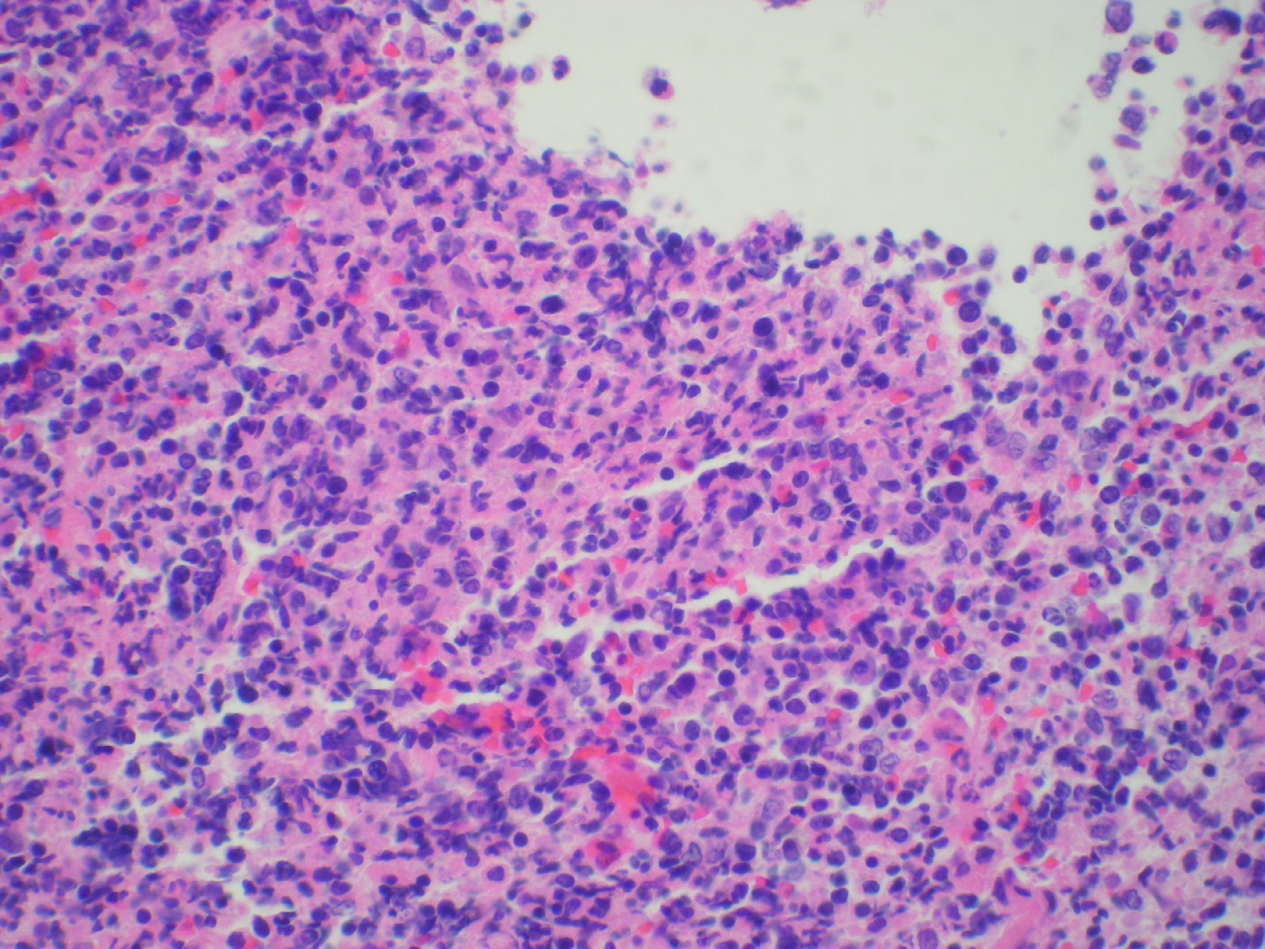
**Gastric biopsy showing atypical lymphoid infiltrate (200 X)**.

**Figure 4:**
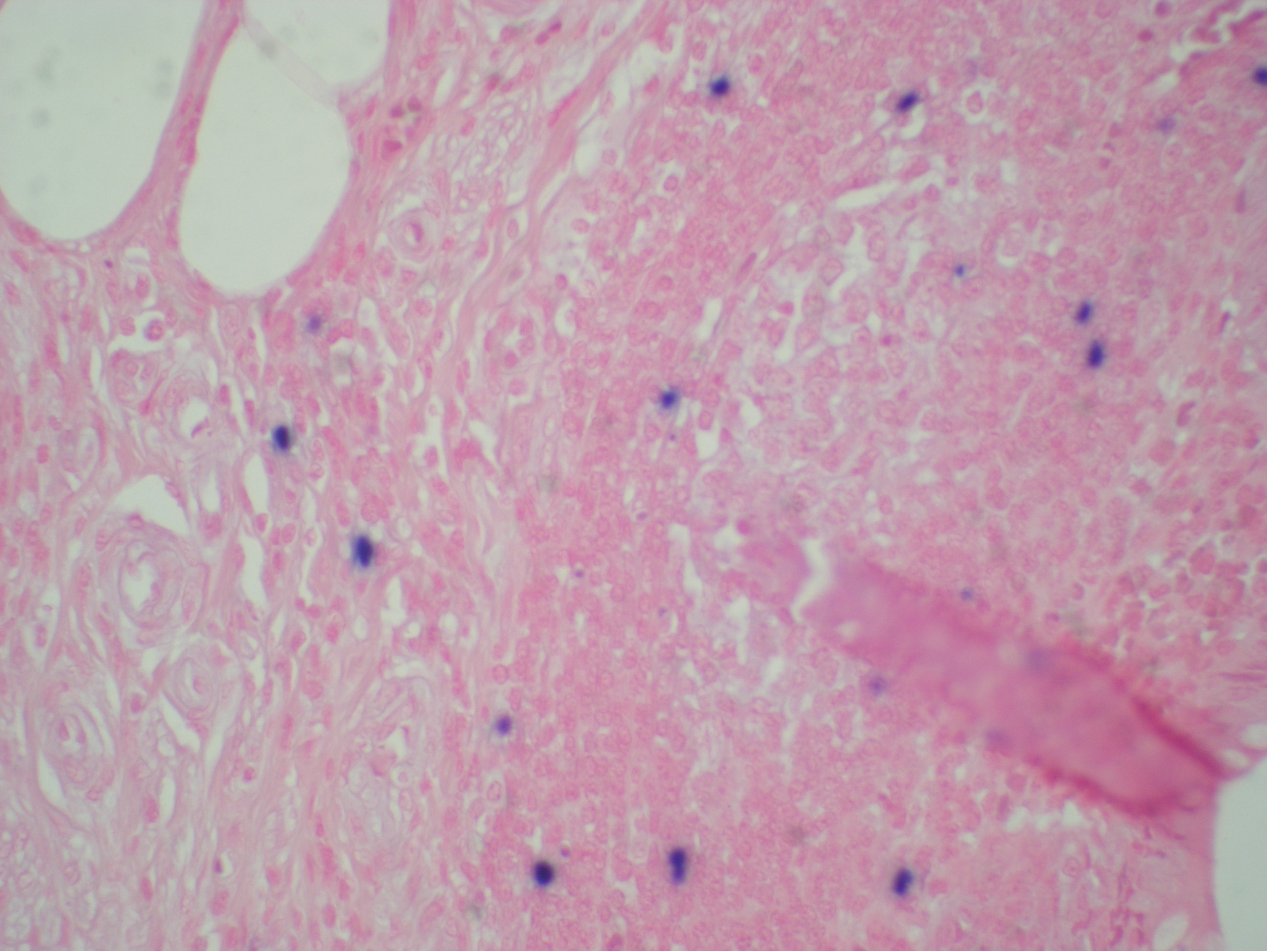
**Appendiceal infiltrate showing scattered Epstein Barr Virus-positive cells (100 X)**.

**Figure 5:**
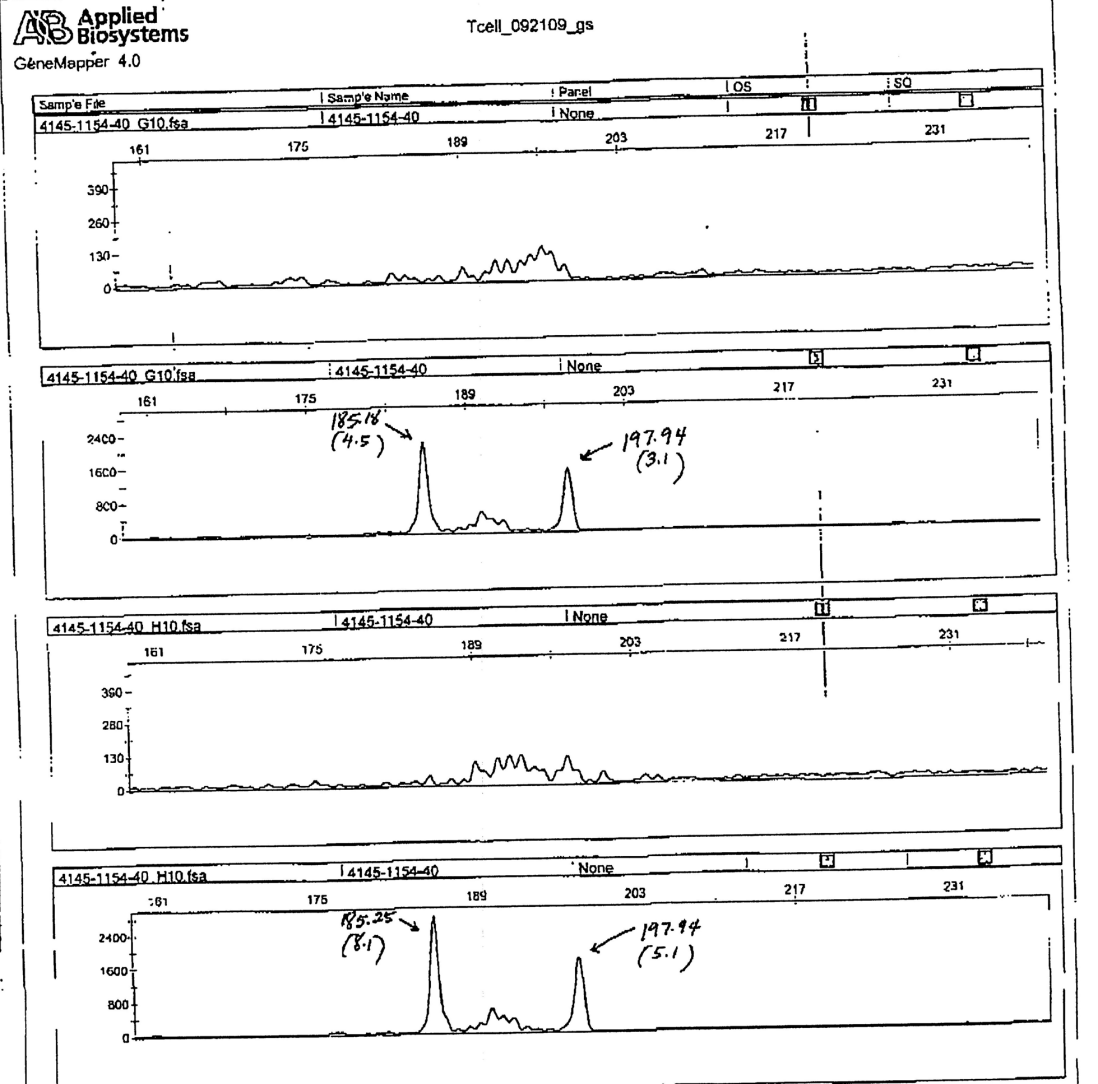
**T cell receptor gene rearrangement by PCR analysis showing monoclonal spike**.

Pathology of his appendix by immunostaining revealed anaplastic cells strongly positive for CD3, CD56 together with strong focal EBV-encoded RNA (EBER) staining (Figures 1, 2, 3 and 4). The malignant cells were negative for CD20, CD30, CD45, CD5, Alk-1 and TCK. The tissue was found to be positive for the T-cell receptor (TCR) by gamma gene rearrangement studies by PCR analysis (Figure 5). Immunoglobulin heavy chain rearrangement (IgH) by PCR analysis did not detect a clonal B-cell population, thereby confirming T-cell lymphoma. A bone marrow examination revealed no involvement with negative flow cytometry and showed normal male karyotype (46, XY). A staging positron emission tomography (PET) scan showed increased radiotracer uptake in the right cervical and left groin lymph nodes along with the 3.3 cm liver mass. Non-specific uptake in the stomach was also observed.

**Figure 6:**
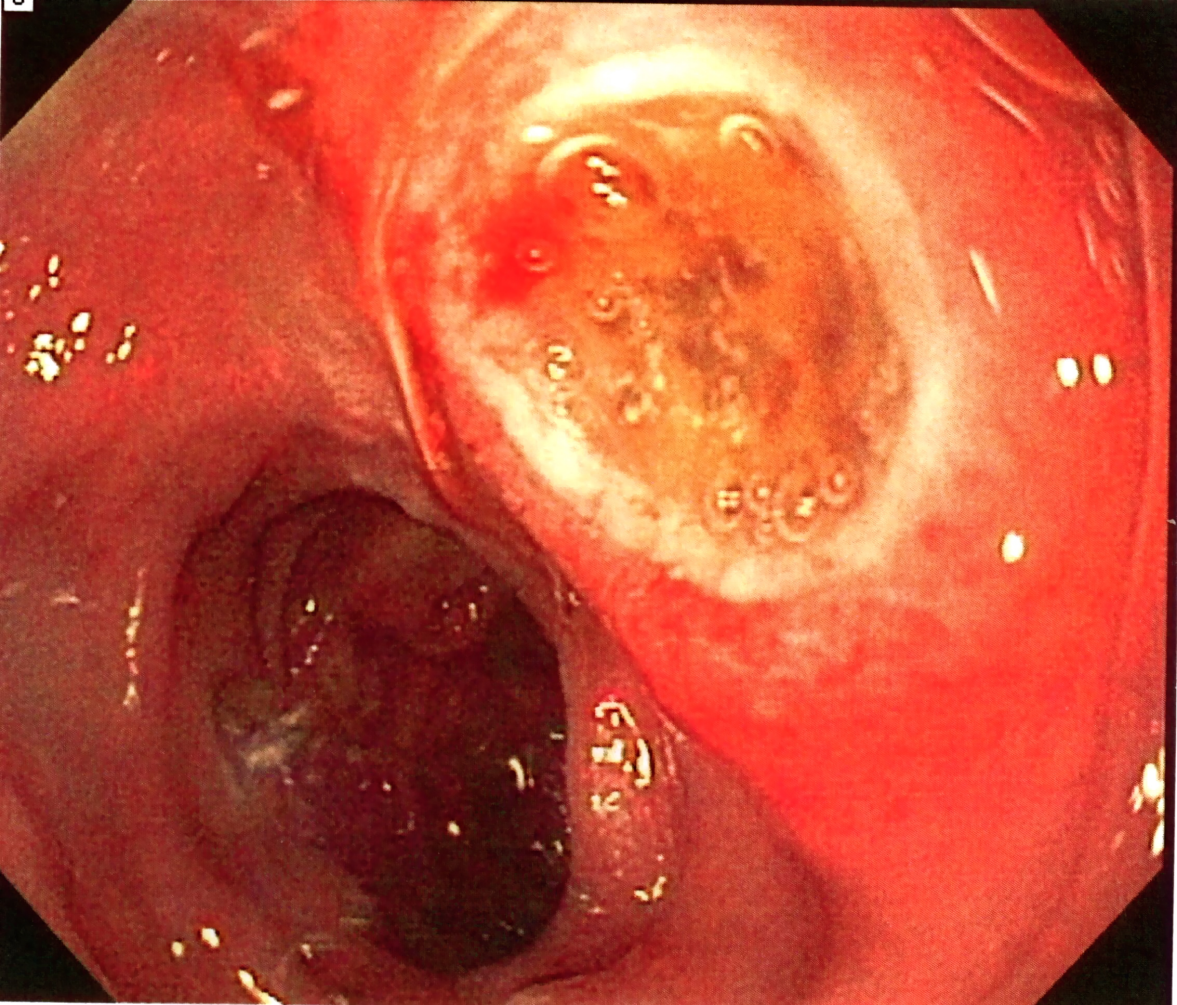
**EGD showing large ulcerated gastric nodule together with large nodules in the duodenum**.

**Figure 7:**
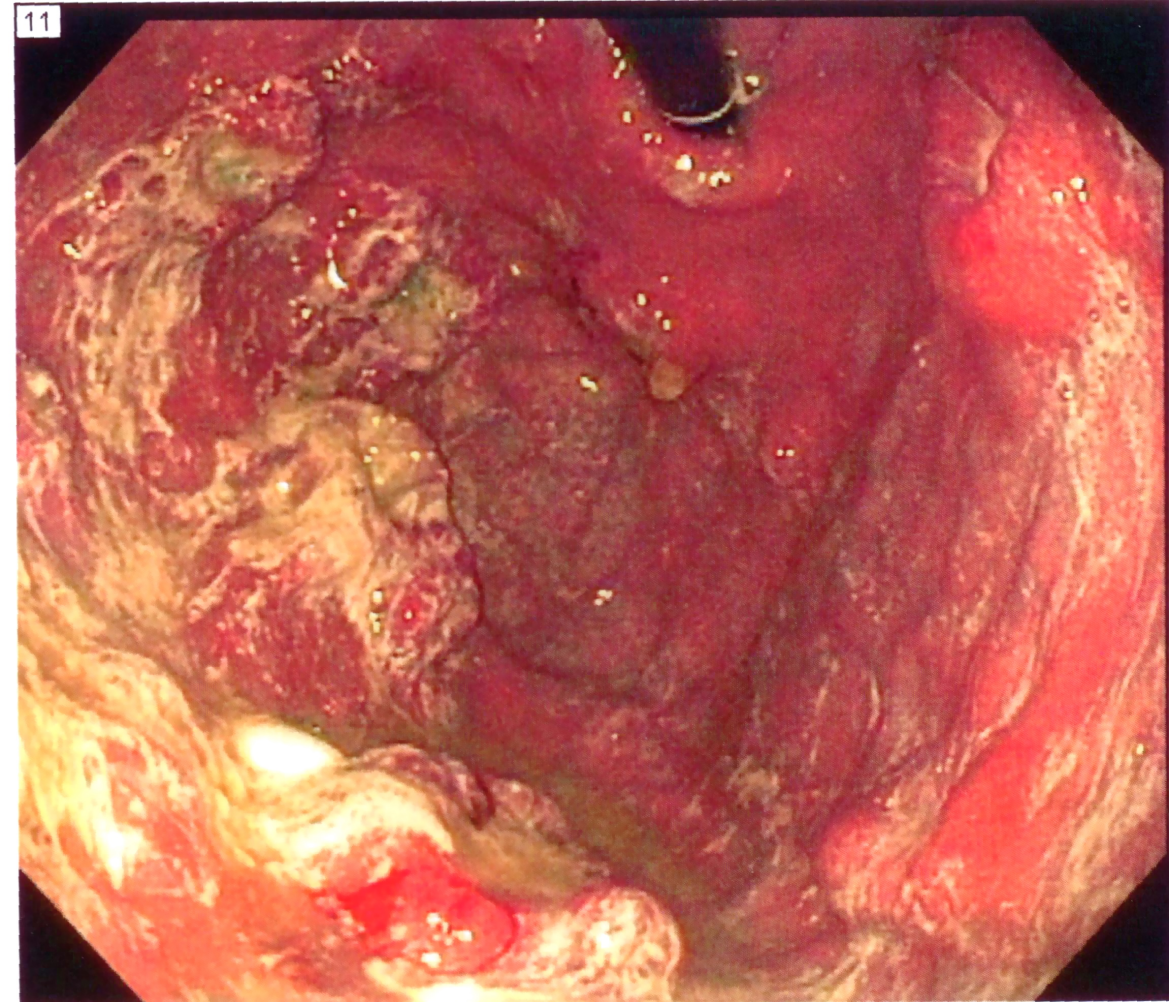
**EGD showing multiple large gastric nodules with central ulceration**.

The patient was re-admitted to the hospital 10 days later, with increasing abdominal pain, symptoms of gastric outlet obstruction, weight loss, headaches and fever. A lumbar puncture was negative for infection or lymphoma. Cranial imaging with a computed tomography (CT) scan was also negative. An esophagogastroduodenoscopy (EGD) was performed revealing multiple ulcerated nodular masses in the stomach and duodenum (Figures 6 and 7). A stomach biopsy gave similar results as the appendix with large anaplastic cells with irregular nuclei. Immunostaining of the gastric specimen confirmed T-cell lymphoma as well as positive EBER staining.

Initial treatment management involved reducing the dose of the patient's immunosuppressive agents and starting chemotherapy. Administration of azathioprine, prednisone and tacrolimus was stopped and low dose sirolimus at 1 mg was given daily. The first cycle of chemotherapy (PEGS) included cisplatin 25 mg/m^2^, etoposide 40 mg/m^2 ^and solumedrol 250 mg administered on days one, two and three, and gemcitabine (Gemzar) 1000 mg/m^2 ^on day one (ongoing Phase II trial SWOG 0350). Our patient had a positive and rapid clinical response to this regimen. Thus, the chemotherapy was changed to a standard CHOP regimen (cyclophosphamide, doxorubicin [Adriamycin], vincristine, prednisone). His gastric outlet obstruction was supported with total parenteral nutrition (TPN) for a few weeks, after which the patient was able to eat well. A repeat PET scan after the second cycle of CHOP showed a significant response.

The main complications during the therapy were pancytopenia, febrile neutropenia and pneumonia. These were managed successfully. He recovered well and is presently receiving treatment as an outpatient. His allo-grafted kidney is also fully functional. Restaging is planned after a total of six cycles of CHOP with a PET scan and EGD.

## Discussion

Our case is interesting due to the latency of PTLD development, the lack of hematological abnormalities and the EBV-positivity, even though the lymphoma was of T-cell origin. The diagnosis became apparent when an appendectomy was performed for abdominal pain. The cytopathology of our patient shows all the typical features of a peripheral T-cell variant of PTLD, where the T-cell lineage of the lymphoma was confirmed by TCR gene rearrangement studies.

While presenting symptoms in the majority of patients are non-specific such as fever and weight loss, approximately 15% of cases present as an emergency with intestinal perforation [5]. Similar to other reports on T-cell type PTLD, which generally occurred more than five years after transplant, this case occurred 17 years after the renal transplant. The high levels of immunosuppression were due to two episodes of graft rejection in the preceding four years.

PTLD can be categorized into three distinct groups based on the World Health Organization classification of lymphoid tissue neoplasms [6] (Table 1). The first group has diffuse B-cell hyperplasia, which is relatively benign and responds well to a reduction in immunosuppression. The second group consists of polymorphic PTLD, which is the most common group in both the adult and pediatric populations. The third group consists of high-grade invasive lymphomas of either T- or B-cell monoclonality. Monomorphic T-cell PTLD is further subdivided into large cell, anaplastic or an unspecified type. While the incidence of T-cell PTLD in renal transplant patients is approximately 15%, a recent study of 21 cases of post-transplant hepatosplenic T-cell lymphoma by Tey *et al. *[7] reported 19 patients who underwent prior renal transplant. This and several other reports [5,7,8] appear to show increased incidence, which might be due to heightened awareness along with use of increasingly potent immunosuppressants.

**Table 1:** The World Health Organization classification of PTLD.

Early Lesions	Polymorphic PTLD	Monomorphic PTLD		
		B-Cell Lymphomas	T-Cell Lymphomas	Other Types

Reactive Plasmacytic Hyperplasia	Polyclonal Monoclonal	• Diffuse large B cell lymphoma • Burkitt/Burkitt-like lymphoma • Plasma cell myeloma	• Peripheral T cell lymphoma • Large CellAnaplastic • Unspecified • Raretypes (gammadelta, Hepatosplenic, T/NKcell)	1. Hodgkin's disease-like 2. Plasmacytoma-like

The etiopathogenesis of T-cell PTLD is not entirely known, although it may be similar to non-Hodgkins lymphoma (NHL) seen in the general population. While a putative role of EBV has been suggested in 89% of cases of B-cell PTLD [9], no direct role for this lymphotropic virus has been confirmed in T-cell PTLD. EBV infects and immortalizes B cells causing unchecked proliferation of EBV-infected cells, as the critical T-cell control of B-cells is lacking in immunosuppressed patients [10]. Human T-cell Lymphotropic Virus 1 (HTLV1) has been reported to cause post renal transplant T-cell lymphomas in Japan [11], due to a higher prevalence of the virus among hemodialysis patients. Our case was unusual in that the lymphoma tested positive for EBV, even though it was of T-cell origin, which is only seen in a small minority of patients [12].

The initial step in treating PTLD is reduction in immunosuppression, and the response is usually seen in three to four weeks, resulting in long term remission in 25% to 63% [13] of adults. Early polyclonal PTLD responds well to a reduction in immunosuppression, whereas monoclonal PTLD generally does not respond to the reduction and has a high mortality rate of 50% to 90% [14]. Early use of anthracycline-based chemotherapy results in long term disease free survival rates of 50% to 60% in monoclonal B-cell lymphomas [15], whereas T-cell PTLD responds very poorly. There are no standardized chemotherapy regimens for PTLD in general and for the T-cell phenotype in particular. Multiple treatment regimens such as CHOP, VAPEC-B (Adriamycin, etoposide, cyclophosphamide, methotrexate, bleomycin and vincristine), anti-IL-6 mAb, bexarotene and antiviral agents have been used with varied results. Other salvage regimens for high-grade lymphomas have also been suggested in the literature [14,15].

## Conclusion

In summary we report the case of a patient with post-renal transplant large T-cell lymphoma, with an unusual presentation of acute appendicitis and EBV positivity. We report successful treatment with chemotherapy and stress the need for heightened awareness of this malignancy in patients with prolonged immunosuppression. Monoclonal T-cell PTLD remains a high mortality disease, and further large multi-centre studies are required to understand the pathogenesis and develop better treatment regimens.

## Consent

Written informed consent was obtained from the patient for publication of this case report and accompanying images. A copy of the written consent is available for review by the Editor-in-Chief of this journal.

## Competing interests

The authors declare that they have no competing interests.

## Authors' contributions

SKR and HZ participated in the conception and literature search. KAM provided and reviewed the pathology slides. SKR, SM, JF and HZ helped to draft the manuscript. All authors read and approved the final manuscript.
